# Esophageal Intramural Pseudodiverticulosis: A Rare Endoscopic Finding

**DOI:** 10.1155/2013/154767

**Published:** 2013-11-19

**Authors:** Luciana Lopes de Oliveira, Fred Olavo Aragão Andrade Carneiro, Elisa Ryoka Baba, Thiago Guimarães Vilaça, Dalton Marques Chaves, Everson Luiz de Almeida Artifon, Eduardo Guimarães Hourneaux de Moura, Paulo Sakai

**Affiliations:** Gastrointestinal Endoscopy Unit, São Paulo University Medical School, 05403-900 São Paulo, SP, Brazil

## Abstract

A 76-year-old woman, presenting with a 4-year history of progressive dysphagia, was submitted to endoscopic examination. The upper endoscopy revealed a proximal esophageal stricture and inflammatory mucosa associated with multiples small orifices in the esophageal wall, some of them fulfilled with white spots suggestive of fungal infection. This was a typical endoscopic finding of esophageal intramural pseudodiverticulosis, a benign and rare condition, related to chronic esophagitis and others comorbid states, such as gastroesophageal reflux disease or infectious esophagitis, diabetes mellitus, alcohol consumption, and achalasia. Dysphagia is the predominant symptom and can be accompanied by esophageal stricture in 80% to 90% of patients. The pathogenesis is unknown, and as the pseudodiverticulosis is an intramural finding, endoscopy biopsies are inconclusive. The main histological finding is dilation of the submucosal glands excretory ducts, probably obstructed by inflammatory cells. The treatment consists in management of the underlying diseases and symptoms relief. In this particular case, the patient was submitted to antifungal drugs followed by endoscopic dilation with thermoplastic bougies, with satisfactory improvement of dysphagia.

## 1. Introduction

Esophageal intramural pseudodiverticulosis (EIP) is a rare condition that consists of small saccular evaginations of the esophageal wall. The pathogenesis is unknown, and it is usually associated with chronic inflammatory conditions, such as diabetes mellitus, esophageal candidiasis, alcohol consumption, achalasia, and gastroesophageal reflux disease [1–3]. 

 EIP affects mainly men over the 6th decade of life [2]. The predominant symptom is dysphagia, present in up to 80% of the patients; however, it can also be asymptomatic or related to chest pain in a minority of cases [4].

 A typical endoscopic finding is the direct visualization of multiples small orifices; however, it is present in a minority of patients. Esophageal barium contrast radiography is more sensible when compared to endoscopy, in which the latter often leads to misdiagnosis with a normal endoscopic view of the esophagus [5, 6]. Others imaging studies, such as chest computed tomography and endoscopic ultrasonography combined with clinical history, are also helpful in the diagnosis of EIP [4, 5]. In most of the cases, histological diagnosis is only possible from surgical specimens, where it is seen dilation of excretory ducts of submucosal glands [4, 7]. 

We report a case of EIP with typical endoscopic findings associated with esophageal stricture, whose response to endoscopic dilation was satisfactory. 

## 2. Case Report

 A 76-year-old female presented with a 4-year history of progressive dysphagia to solids. She had diabetes and hypertension as comorbidities. One year after the onset of dysphagia, she was diagnosed with a multinodular thyroid goiter and received surgical treatment. As the symptoms persisted, she was referred to upper endoscopy unit. 

**Figure 1:**
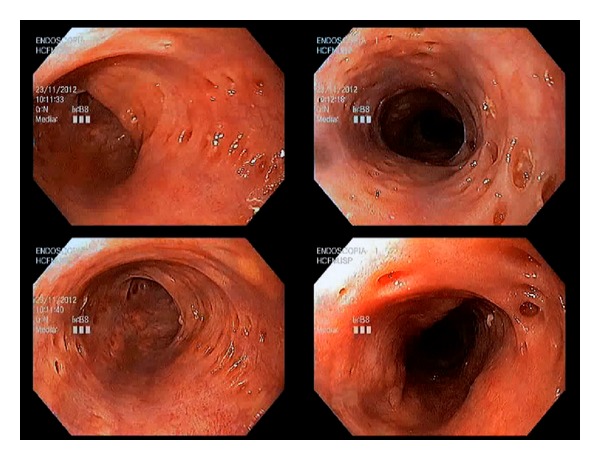
Endoscopic view of esophageal intramural pseudodiverticulosis revealing several small orifices, measuring between 2 and 4 mm in diameter.

 The endoscopic examination revealed a proximal esophageal stricture and several small orifices, measuring between 2 and 4 mm in diameter, with different depths, some of them filled by whitish granules, which biopsy demonstrated fungal infection (Figure 1). This typical endoscopic finding allowed the diagnosis of EIP associated with *Candida* esophagitis. 

**Figure 2:**
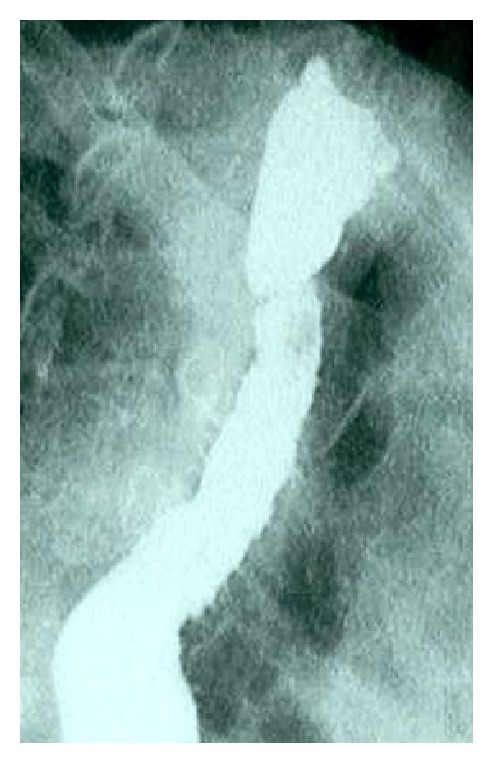
Esophageal barium contrast radiography with a proximal esophageal stricture and small areas of contrast enhancement parallel to the esophageal wall, compatible with esophageal intramural pseudodiverticulosis.

Esophageal barium contrast radiography confirmed the diagnosis of proximal esophageal stricture, as well as the identification of small areas of contrast enhancement parallel to the esophageal wall, a characteristic feature of EIP (Figure 2). 

The patient was treated for the fungal infection and referred to endoscopic dilation of the proximal stricture. She received one dilation session with thermoplastic bougies, whose response was clinically significant. 

## 3. Discussion

 EIP is a rare condition with unknown etiology, and the predominant anatomic finding is an excretion impairment of submucosal glands leading to ductal dilation, with the consequent formation of pseudodiverticula [4, 5, 7]. Some authors reported that obstruction and subsequent dilation of excretory ducts were caused by inflammatory cells, epithelial desquamation, submucosal fibrosis, or a combination of these factors, as occurred in gastroesophageal reflux disease or infectious esophagitis [4, 8]. Otherwise, esophageal intramural pseudodiverticulosis is also related with diabetes mellitus, achalasia, and other motor disorders of the esophageal wall, which may lead to ductal dilation by myoepithelial cells dysfunction that are responsible for duct contraction and glandular excretion [7, 9, 10].

 Dysphagia is the most common symptom in these patients, and due to its evolution and widespread utilization, an upper endoscopy is usually the first diagnostic procedure performed in these patients. In this examination, a direct visualization of small orifices in esophageal lumen allows the diagnosis of EIP; however, according to literature, it is only seen in 20% of the cases [4, 8]. Since the lesions are intramural, endoscopic biopsies usually demonstrate nonspecific acute or chronic esophagitis. In most of the cases, histological diagnosis is only possible from surgical specimens, where it is seen dilation of the excretory ducts of submucosal glands [4, 7].

 Esophageal contrast radiography is a more sensitive diagnostic method, where narrow ostia and small evaginations are observed in esophageal wall with continuity to its lumen [5, 7]. Computed tomography is characterized as a diffuse thickening of the esophageal wall with intramural gas and diffuse irregularities in the organ lumen [1, 5, 6]. In cases where an endoscopic ultrasound is necessary, it can be observed multiple hyperechoic images in esophageal wall that correspond to the intramural gas. 

 EIP is a benign condition, and stricture is its main complication. Esophageal stricture is described in up to 80% to 90% of cases, but dysphagia occurs irrespectively of this finding [4, 8]. Some authors described a higher prevalence of esophageal cancer in this disease; however, there is no literature evidence that supports it as premalignant condition [4]. Complications such as esophageal fistula or perforation are rare [1, 8].

The treatment consists of the management of comorbidities and the underlying esophagitis, with measures for gastroesophageal reflux disease, alcohol withdrawal, and antibiotic treatment for associated infections [2, 4, 5, 11]. In patients with strictures, it is reported that endoscopic dilation provides highly effective improvement in symptoms [1, 6, 10]. However, in long-term followup, it is not observed a regression of the pseudodiverticula, which persist despite clinical improvement and appropriate treatment [5, 8].
